# Vasoreactivity of the optic nerve head, nailfold, and facial skin in response to cold provocation in normal-tension glaucoma patients

**DOI:** 10.1186/s12886-023-03059-0

**Published:** 2023-07-12

**Authors:** Nana Takahashi, Naoki Kiyota, Hiroshi Kunikata, Mai Yamazaki, Takayuki Nishimura, Yukihiro Shiga, Hisae Aoyagi, Miwako Shidomi, Tomohiro Tsuda, Toshihiko Ohtsuka, Takahiro Tomida, Toru Nakazawa

**Affiliations:** 1 Department of Ophthalmology, Tohoku University Graduate School of Medicine, Miyagi, Japan; 2 Department of Retinal Disease Control, Tohoku University Graduate School of Medicine, Miyagi, Japan; 3 Seiryo Eye Clinic, Miyagi, Japan; 4 Department of Health Science Research Planning Division, Rohto Pharmaceutical Co., Ltd, Osaka, Japan; 5 Department of Internal Medicine and Food Development Division, Rohto Pharmaceutical Co., Ltd, Osaka, Japan; 6 Department of Advanced Development, Casio Computer Co., Ltd, Tokyo, Japan; 7 Department of Ophthalmic Imaging and Information Analytics, Tohoku University Graduate School of Medicine, Miyagi, Japan; 8 Department of Advanced Ophthalmic Medicine, Tohoku University Graduate School of Medicine, Miyagi, Japan

**Keywords:** Ocular blood flow, Laser speckle flowgraphy, Autoregulation, Vasoreactivity

## Abstract

**Background:**

The dysfunction of optic nerve head (ONH) hemodynamics has been suggested to be involved in the pathogenesis of normal-tension glaucoma (NTG). The aim of this study was to compare vasoreactivity in the ONH, nailfold, and facial skin in response to cold-water provocation in NTG patients and healthy controls.

**Methods:**

We performed cold-water provocation in 14 eyes of 14 NTG patients and 15 eyes of 15 age-matched control subjects. Laser speckle flowgraphy-derived tissue-area mean blur rate (MT), skin blood flowmetry-derived pulse wave amplitude (PA), nailfold capillaroscopy-derived nailfold capillary diameter, and other clinical parameters were recorded at baseline and 4 and 6 min after the cold stimulus. We compared changes (as percentages) in these variables in the NTG and control subjects with a linear mixed-effects model and evaluated correlations between these changes with Spearman’s rank correlation coefficient.

**Results:**

The interaction term between the NTG group (reference, control group) and the 4-min protocol step (reference, baseline) significantly affected the changes in MT, nailfold capillary diameter and PA (β = -9.51%, *P* = 0.017, β = -20.32%, *P* = 0.002; β =  + 18.06%, *P* = 0.017, respectively). The change in MT was positively correlated with the change in nailfold capillary diameter, and negatively correlated with the change in PA (*r* = 0.39, *P* = 0.036; *r* = -0.40, *P* = 0.031, respectively).

**Conclusion:**

NTG patients showed abnormal vasoconstriction in the ONH and nailfold and vasodilation in the facial skin in response to cold-water provocation.

**Supplementary Information:**

The online version contains supplementary material available at 10.1186/s12886-023-03059-0.

## Background

Glaucoma, the second most common cause of blindness worldwide [1, 2], is characterized by progressive retinal ganglion cell death and associated visual field loss [3]. High intraocular pressure (IOP) is the only modifiable risk factor for primary open-angle glaucoma, but the existence of normal-tension glaucoma (NTG) indicates that high IOP is not the only risk factor for glaucoma development or progression. One suspected non-IOP risk factor is chronic blood flow (BF) impairment in the optic nerve head (ONH), which may be associated with abnormal vasoreactivity in response to external stimuli (e.g., the vasospastic response to cold stimulus) [4, 5].

Cold provocation testing (CPT) has been used to evaluate the role of vascular dysregulation in glaucoma in several studies. This technique elicits vasoconstriction by activating the sympathetic autonomic nervous system [6–8]. Most previous studies have combined CPT with nailfold capillaroscopy and reported excessive nailfold capillary constriction during testing in glaucoma patients [9–11]. The popularity of these methods may be due to the ease of access to the nailfold and suggestions that there is a relationship between systemic abnormal vasoreactivity, such as Raynaud’s phenomenon, and NTG [12, 13]. In addition, excessive constriction of nailfold capillaries following CPT has been found to be associated with faster visual-field defect progression in primary open-angle glaucoma patients [14]. This suggests that there may be a clinically significant relationship between BF in the nailfold capillaries and the eye. Nevertheless, there have been few reports in the field of ophthalmology that have investigated the vasospastic response of glaucoma patients in the retina, the rim of the ONH, and the retrobulbar region during CPT [15–17]. Thus, there is an unfortunate lack of data on the vasoreactive response to cold provocation in the deep areas of the ONH, which is believed to be the primary site of glaucomatous lesions. Against this background, we chose to use laser speckle flowgraphy (LSFG), an easy and reproducible method of measuring BF in the deep area of the ONH [18, 19], in addition to a nailfold capillaroscopy device [20, 21].

We also went a step further and examined facial skin BF dynamics, using a skin blood flowmetry device, because the vascular response to cold provocation (i.e., sympathetic stimulation) may be different in this region [22] and because the face is even more accessible than the nailfold or deep areas of the ONH. Unfortunately, we lack multimodal imaging data obtained during CPT. In fact, it remains unclear which area in the body is most suited for detecting abnormal vasoreactivity in NTG patients and which device would be most suitable for obtaining such data. Precise and reproducible measurements of regional differences in the response of the vasculature to CPT may lead to further understanding of the vascular component in glaucoma pathogenesis and the association with clinical findings. Thus, this study sought to establish a CPT protocol and compare vasoreactivity in NTG and healthy controls during CPT in the deep ONH, nailfold, and facial skin with recently developed imaging devices, including LSFG, nailfold capillaroscopy, and skin blood flowmetry, respectively.

## Materials and methods

### Subjects

This prospective study comprised 14 eyes of 14 NTG patients and 15 eyes of 15 control subjects who visited the Seiryo Eye Clinic, located in Miyagi, Japan, between August and November 2021. Informed consent was obtained from the subjects before the CPT was performed. This study followed the tenets of the Declaration of Helsinki and was approved by the Ethics Committee of Tohoku University School of Medicine (protocol number 2021–1-434). When both eyes satisfied the inclusion criteria, the left eye was selected for the analysis. NTG was diagnosed by a glaucoma specialist (N.T.).

The inclusion criteria for both groups were (1) age between 30 and 75 years, (2) cataract grade less than or equal to 2 on the Emery-Little classification [23], and (3) IOP ≤ 21 mmHg. An additional inclusion criterion for the control group was normal findings in slit-lamp, fundoscopy, and optical coherence tomography examinations. Additional inclusion criteria for the NTG group were (1) the presence of glaucomatous optic disc changes with corresponding visual field (VF) defects matching the Anderson-Patella criteria [24], (2) abnormally reduced circumpapillary retinal nerve fiber layer thickness (cpRNFLT), (3) a normal, open angle in a gonioscopic examination, and (4) mean deviation (MD) > -12 dB.

Subjects were excluded if they (1) had a history of ocular or systemic disease causing optic nerve damage, (2) had high myopia (axial length [AL] > 26.5 mm or spherical equivalent < -8 diopters), (3) had a history of intraocular surgery, (4) had a history of smoking within the previous 4 years, (5) were pregnant, or (6) had a history of discomfort during any kind of cold provocation. All subjects abstained from alcohol and caffeine for at least 6 h before the cold-water provocation.

### Measurement of baseline clinical characteristics

Baseline clinical variables were measured before the CPT. Logarithmic minimum angle of resolution (logMAR) was used to represent best-corrected visual acuity (BCVA). IOP was measured with Goldmann applanation tonometry, AL was measured with the OA-2000 (Tomey Corporation, Aichi, Japan), cpRNFLT was measured with swept-source optical coherence tomography (DRI OCT; Triton, Topcon, Inc., Tokyo, Japan), and the VF was measured with the 24–2 program of the Humphrey Field Analyzer (Carl Zeiss Meditec, Dublin, CA, USA). Reliable results were obtained from all NTG subjects in VF testing (fixation errors < 20%, false positives < 33%, and false negatives < 33%) and OCT examinations (signal strength > 60). Blood pressure (BP) and pulse rate (PR) were conventionally measured in the brachial artery at the height of the heart (HBP-1300; Omron Colin Co., Ltd., Tokyo, Japan). Pupil dilation was performed with 0.4% tropicamide (Mydrin M; Santen Pharmaceutical Co., Ltd., Osaka, Japan). Mean blood pressure (MBP) and mean ocular perfusion pressure (MOPP) were calculated as follows: MBP = diastolic blood pressure (DBP) + 1/3 (systolic blood pressure [SBP]—DBP); MOPP = 2/3 MBP – IOP.

### LSFG measurements and nailfold capillary imaging

ONH BF was measured with LSFG. The principles of LSFG have previously been described in detail [25]. Briefly, this device measures the pattern of speckle contrast produced by the interference of a laser scattered by blood cells moving in the blood vessels. This study used the LSFG-NAVI device (Softcare Co., Ltd., Fukutsu, Japan). Mean blur rate (MBR), which is known to represent BF velocity, is the main LSFG variable and is expressed in arbitrary units. MBR images of the fundus are acquired continuously at the rate of 30 frames per second over a 4-s period and then averaged to produce a composite map of ocular BF. The accompanying analysis software can automatically divide the MBR map into the large vessel and tissue (capillary) areas of the ONH and determine specific values for each. The focus of this study was on tissue-area MBR (MT), because MT has been reported to represent BF in the deeper regions of the ONH [18, 19].

Nailfold capillaries were observed under a light microscope (Kekkan-Bijin; At Co., Ltd., Osaka, Japan) after application of mineral oil to reduce light reflection, as previously described [20, 21]. The observations were performed at the fourth finger of the left hand [26–28]. Capillary images were captured using the Capillary Analysis System (At Co., Ltd.). The images of the nailfold capillaries were numerically analyzed for their average length and diameter [20, 29].

### Casio blood flowmetry, a novel method of measuring skin BF

Facial skin BF was measured with a skin blood flowmeter (Casio Computer Co., Ltd., Tokyo, Japan). This measurement is completed by simply placing the camera on the measurement site (in this study, the skin of the left cheek) for 30 s. Luminance data can be acquired from the image sensor 30 times per second. The principle of operation of this blood flowmeter is to measure skin BF via pulse wave extraction from the captured images, represented by pulse wave amplitude (PA). Hemoglobin in skin capillaries absorbs green light, and the luminance of the green component of light reflected by the skin thus changes with flux and reflux of skin blood perfusion. By capturing changes in the luminance of the minute green component of this signal with an image sensor, time-series data for luminance can be extracted.

As the skin blood flowmeter is a novel device for measuring skin BF, we first confirmed its reproducibility in this study. For this, we independently enrolled 12 healthy volunteers (45.6 ± 11.0 years, male to female ratio = 6:6). All subjects abstained from alcohol and caffeine for at least 6 h before the measurements. As shown in Supplementary Figure S1, at an initial visit, two sets of skin blood flowmeter measurements were obtained consecutively, at 10:00 a.m. (sets 1 and 2). At a second visit (between 1 to 7 days later), skin blood flowmeter measurements were again obtained at 10:00 a.m. (set 3), the examiner was changed, and a final measurement was obtained (set 4). The volunteers took 5-min rests between set 1 and set 2 and between set 3 and set 4. The coefficient of variation (CV) for the PA values was calculated to investigate the intrasession reproducibility (for sets 1 and 2), intersession reproducibility (for sets 1 and 3), and interexaminer reproducibility (for sets 3 and 4), following a method in a previous report [30]. We also checked whether skin blood flowmeter-derived PA correlated with laser speckle perfusion imager-derived BF, which has previously been used as a parameter to measure BF [31–34] in the same facial skin region.

### Cold-water provocation test

The cold-water provocation test was performed as previously described [15, 16], between 1:00 and 5:00 p.m. in a dark, quiet room. The room temperature was kept at 20 °C. We prepared warm water at 40 °C and cold water at 4 °C. The cold provocation testing comprised 5 steps: baseline, warm water application, cold water application, 4-min post-cold immersion measurements, and 6-min post-cold immersion measurements. LSFG-derived parameters, BP, PR, skin blood flowmetry-derived PA, nailfold capillary diameter, and IOP were measured at baseline, 4 min, and 6 min. After the baseline examination, the subjects immersed their right hands into the warm water for 2 min, and then immediately immersed the same hand into the cold water for 1 min. The percentage changes in MT, PA, and nailfold capillary diameter versus baseline were calculated as follows:

% Change in X (t) = (X (t)—X (baseline)) / X (baseline).

(X = MT, PA, nailfold capillary diameter; t = baseline, 4 min, or 6 min).

**Fig. 1:**
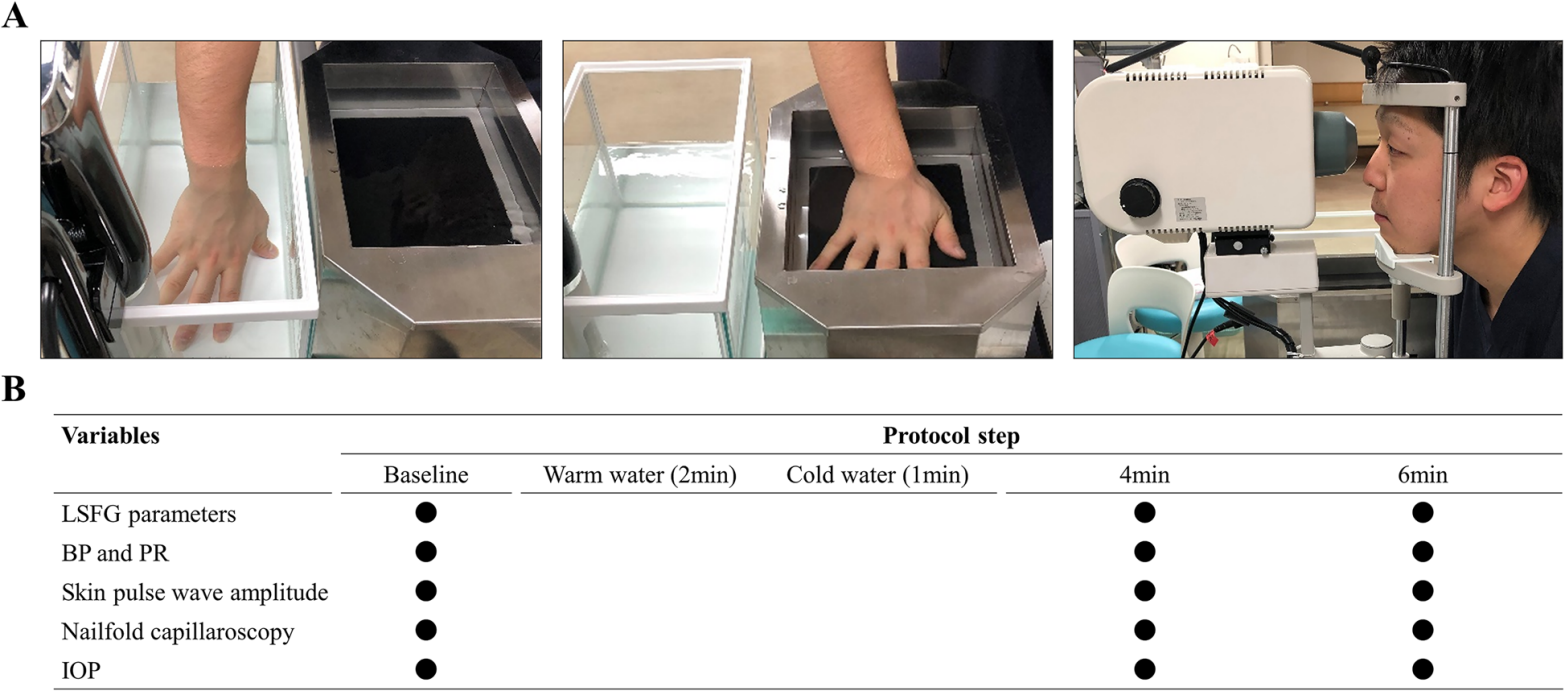
**A** Representative photographs of our testing protocol. From left to right: photographs of a subject immersing his right hand into 40 °C warm water and 4 °C cold water and undergoing LSFG measurement. **B** Testing protocol with five steps: baseline, warm water (2 min), cold water (1 min), and 4 min and 6 min after the cold-water immersion. During the test, LSFG measurements, BP, PR, skin pulse wave amplitude, nailfold capillaroscopy diameter, and IOP were recorded. The black circles represent measurement time points. Baseline LSFG measurements were repeated three times and averaged

**Fig. 2:**
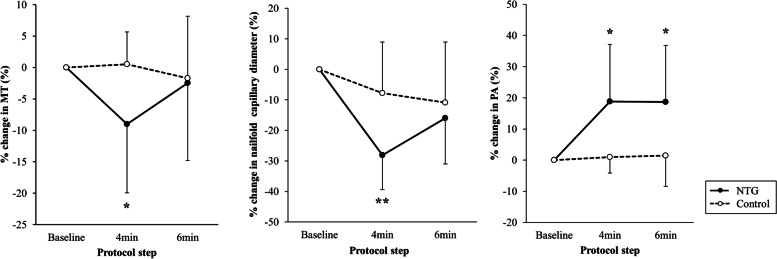
Differences in the vascular response to cold-water provocation. The x-axis shows the protocol steps, and the y-axis shows the percentage change in the indicated parameters. Black circles with straight lines indicate variables from the NTG group and white circles with dotted lines indicate variables from the control group. The asterisks indicate the statistical significance of the interaction term between the indicated group and protocol step (linear mixed-effects model, *P < 0.05, **P < 0.01)

X (t) indicates the value of MT, PA, or nailfold capillary diameter at each protocol step. Figure 1 shows representative images and the protocol schema. It was possible to complete each measurement, including MT, PA, nailfold capillary diameter, IOP, and BP/PR, within 30 s. Some measurements, such as BP, can be done concurrently with other tests. Thus, with efficient use of time and technique by the three experienced examiners, it was generally possible to complete a single time-point test in less than a minute. There were no instances where the test duration exceeded two minutes and overlapped with the next measurement time point. The protocol steps at which each parameter reached its peak (Fig. 2), calculated as the percentage changes in MT, nailfold capillary diameter, and PA, occurred at the 4,4,6-min protocol steps, respectively.

### Statistical analysis

All data are shown as the mean ± standard deviation. Wilcoxon’s rank-sum test or Fisher’s exact test were used for group comparisons (i.e., control vs. NTG group). Multiple name-logistic regression analysis was used for group comparisons adjusting for potential confounding factors. An analysis of variance of linear mixed-effects model was used to see whether there was any change in BP, PR, IOP, or MOPP during the provocation testing, setting the “subject variable” as a random effect. A linear mixed-effects model was used to determine the statistical difference in percentage changes in MT, PA, and nailfold capillary diameter, setting the interaction term between “protocol step” (reference, baseline) and “group” (reference, control group) as fixed effects and setting the “subject variable” as a random effect. Spearman’s rank correlation coefficient was used to determine the correlation between the percentage change in MT and percentage change in nailfold capillary diameter or percentage change in PA, and between PA and laser speckle perfusion imager-derived BF parameters. We calculated the CV for PA to confirm its intrasession reproducibility, intersession reproducibility, and interexaminer reliability. All statistical analyses were performed with R software (version 3.2.5). The significance level was set at *P* < 0.05.

## Results

### Reproducibility of skin blood flowmetry-derived PA

Intrasession reproducibility, intersession reproducibility, and interexaminer reliability for PA were relatively high: the CV values were 7.3% ± 6.5%, 12.5% ± 7.0%, and 6.5% ± 4.8%, respectively. PA and laser speckle perfusion imager-derived BF showed a statistically significant correlation (*r* = 0.49, *P* = 0.007).

### Comparison of clinical characteristics between groups

As shown in Table 1, lower cpRNFLT, lower MD, shorter nailfold capillary length, and thinner nailfold capillary diameter were observed in the NTG group (105.2 ± 13.7 vs. 75.3 ± 14.2, *P* < 0.001; -0.8 ± 1.2 vs. -3.0 ± 2.5, *P* = 0.003; 977.8 ± 265.4 vs. 592.9 ± 374.4, *P* = 0.004; and 29.0 ± 7.0 vs. 23.5 ± 5.1, *P* = 0.036, respectively). Other variables did not reach statistical significance (*P* = 0.087 – 0.930). Because of the multicollinearity between nailfold capillary length and nailfold capillary diameter, we created two multiple name-logistic regression models in which IOP, age, gender, and nailfold capillary diameter or nailfold capillary length were included as explanatory variables and the “group” variable (reference, control group) as the response variable. In model 1, nailfold capillary length was significantly shorter in the NTG group (0.992 [0.987—0.998], *P* = 0.009). In model 2, thinner nailfold capillary diameter was associated with the NTG group (0.754 [0.595—0.956], *P* = 0.020).

**Table 1:** The comparison of clinical characteristics between the groups

**Variables**	**Controls**	**NTG**		**Model 1**		**Model 2**	
	(*n* = 15 eyes)	(*n* = 14 eyes)	*P* value	OR (95% CI)	*P* value	OR (95% CI)	*P* value
BCVA, logMAR	-0.12 ± 0.09	-0.10 ± 0.10	0.552				
Axial length, mm	24.7 ± 1.3	25.0 ± 1.1	0.678				
Intraocular pressure, mmHg	14.1 ± 2.5	12.2 ± 3.0	0.087	1.155 (0.844-1.580)	0.369	0.930 (0.687-1.260)	0.642
CpRNFLT, μm	105.2 ± 13.7	75.3 ± 14.2	< 0.001				
MT, AU	11.9 ± 1.7	11.1 ± 3.3	0.527				
Mean deviation, dB	-0.8 ± 1.2	-3.0 ± 2.5	0.003				
Subject age, years	54.5 ± 6.0	57.7 ± 12.6	0.294	1.247 (1.027-1.515)	0.026*	1.139 (1.002-1.295)	0.047*
Male (ref, female)	8	10	0.313†	0.162 (0.012-2.170)	0.169	0.271 (0.036-2.067)	0.208
BMI, kg/m2	22.5 ± 4.3	23.3 ± 2.7	0.260				
Diabetes mellitus, n	1	3	0.241†				
Dyslipidemia, n	2	5	0.122†				
Hypertension, n	3	5	0.343†				
Systolic BP, mmHg	131.9 ± 19.9	132.6 ± 24.5	0.527				
Diastolic BP, mmHg	87.9 ± 12.1	85.9 ± 10.3	0.237				
Mean BP, mmHg	102.6 ± 14.5	101.1 ± 14.6	0.930				
MOPP, mmHg	54.6 ± 9.9	53.2 ± 10.4	0.678				
Pulse rate, bpm	70.9 ± 8.5	69.0 ± 10.5	0.743				
Nailfold capillary length	977.8 ± 265.4	592.9 ± 374.4	0.004	0.992 (0.987-0.998)	0.009*		
Nailfold capillary diameter	29.0 ± 7.0	23.5 ± 5.1	0.036			0.754 (0.595-0.956)	0.020*
Prostaglandin analogs, n (%)	-	10 (71.4)	-				
β-antagonists, n (%)	-	7 (50.0)	-				
Carbonic anhydrase inhibitors, n (%)	-	2 (14.3)	-				

*AU* arbitrary unit, *BCVA* best corrected visual acuity, *BMI* body mass index, *bpm* beats per minute, *BP* blood pressure, *CpRNFLT* circumpapillary retinal nerve fiber layer thickness, *logMAR* logarithmic minimum angle of resolution, *MOPP* mean ocular perfusion pressure, *MT* tissue area mean blur rate

Wilcoxon's test was used for group comparison

^†^ Fisher’s exact test

^*^ indicate the statistical significance in multiple name-logistic regression analysis

### Changes in systemic variables during the CPT

No adverse events were observed during the provocation. As shown in Table 2, IOP increased during the CPT in the control group (*P* = 0.007); however, no systemic variables, including SBP, DBP, PR, MBP, and MOPP, changed significantly in either group (*P* = 0.086 – 0. 880).

**Table 2:** Changes in systemic variables during the cold provocation test

**Variables**	**Controls, *n* = 15**				**NTG, *n* = 14**			
	**Baseline**	**4 min**	**6 min**	***P* value**	**Baseline**	**4 min**	**6 min**	***P* value**
SBP, mmHg	131.9 ± 20.1	135.9 ± 23.1	132.5 ± 22.0	0.104	131.5 ± 25.5	132.1 ± 22.7	133.4 ± 21.4	0.646
DBP, mmHg	87.9 ± 12.1	89.5 ± 12.5	87.6 ± 12.7	0.261	85.9 ± 10.3	85.5 ± 12.2	83.8 ± 11.6	0.486
IOP, mmHg	13.8 ± 2.8	15.0 ± 3.1	14.5 ± 3.0	0.007*	14.2 ± 3.6	14.3 ± 3.5	14.5 ± 3.1	0.704
PR, bpm	70.9 ± 8.5	72.9 ± 8.0	70.9 ± 7.3	0.086	69.0 ± 10.5	67.9 ± 10.5	67.3 ± 11.2	0.276
MBP, mmHg	102.6 ± 14.5	105.0 ± 15.6	102.6 ± 15.3	0.126	101.1 ± 14.6	101.0 ± 14.6	100.3 ± 13.8	0.880
MOPP, mmHg	54.6 ± 9.1	55.0 ± 9.9	53.9 ± 9.6	0.465	53.2 ± 10.4	53.1 ± 11.0	52.4 ± 10.2	0.767

*bpm* beats per minute, *DBP* diastolic blood pressure, *IOP* intraocular pressure, *MBP* mean blood pressure, *MOPP* mean ocular perfusion pressure, *PR* pulse rate, *SBP* systolic blood pressure

*P* value: linear mixed-effects model

*indicate the statistical significance in an analysis of the variance of the linear mixed-effects model

### Differences in the vascular response to cold provocation

As shown in Fig. 2, there was a statistically significant interaction term between the NTG group (reference, control group) and the 4-min protocol step (reference, baseline) against the percentage in change in MT (β = -9.51%, *P* = 0.017). There was a statistically significant interaction term between NTG group and the 4-min protocol step against percentage change in nailfold capillary diameter (β = -20.32%, *P* = 0.002). There were statistically significant interaction terms between the NTG group and the 4-min protocol step and the NTG group and 6-min protocol step against percentage change in PA (β =  + 18.06%, *P* = 0.017; β =  + 17.72%, *P* = 0.018, respectively).

### Relationship among vasoreactivity parameters

**Fig. 3:**
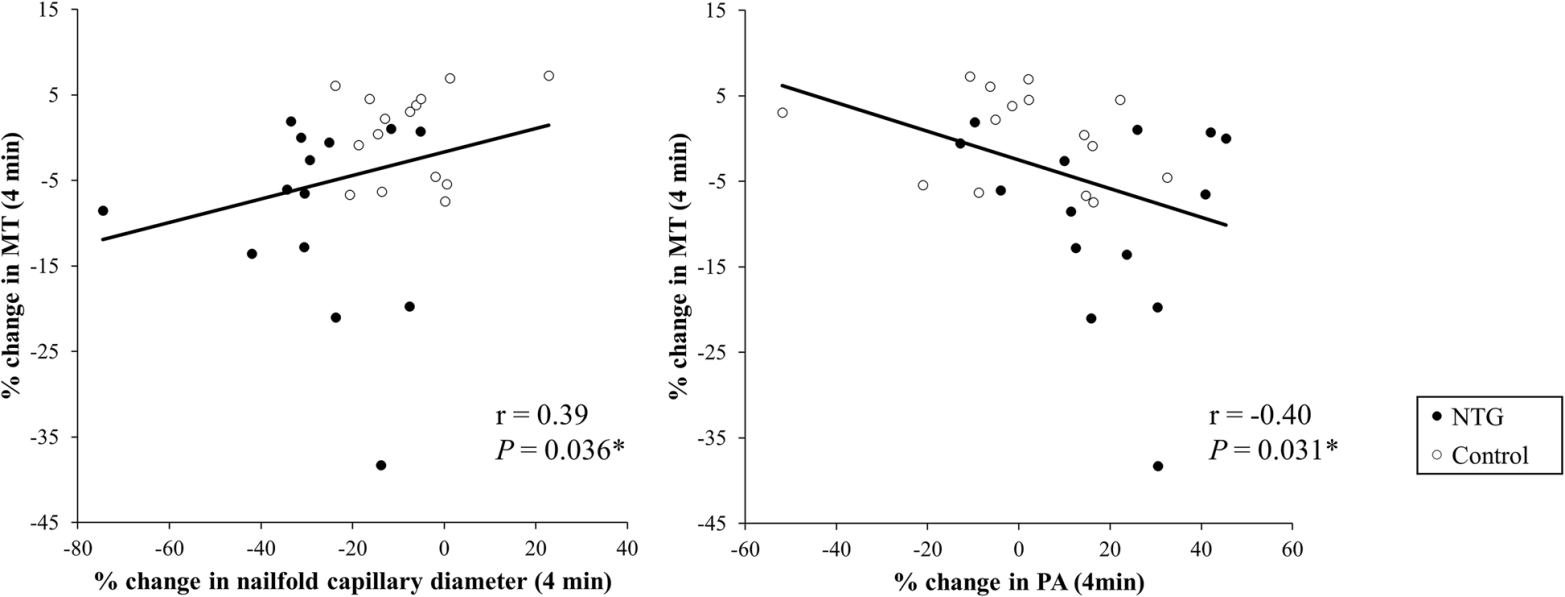
The relationship between percentage change in MT and percentage change in PA or nailfold capillary diameter. The percentage change in MT was significantly correlated with the percentage changes in nailfold capillary diameter and in PA in the overall subjects (*r* = 0.39, *P* = 0.036; *r* = -0.40, *P* = 0.031, respectively)

As shown in Fig. 3, the percentage change in MT was significantly positively correlated with the percentage change in nailfold capillary diameter (*r* = 0.39, *P* = 0.036) and negatively with the percentage change in PA (*r* = -0.40, *P* = 0.031) in the overall subjects.

### Comparative visualization of healthy subject and NTG patient: LSFG images, nailfold capillary diameter and skin pulse wave alterations

**Fig. 4:**
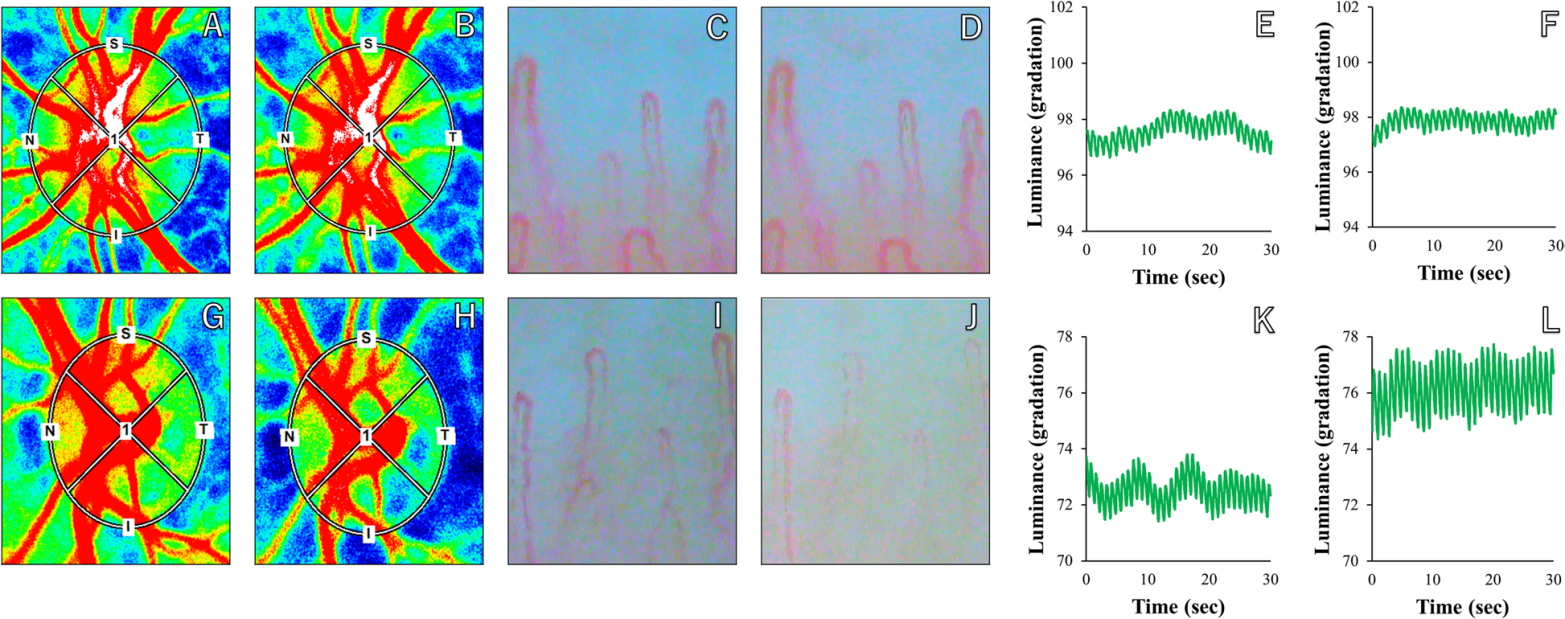
Representative LSFG images, nailfold capillary diameter images, and graphs showing skin pulse wave alteration during the protocol. **A**-**F** The upper panels show a healthy subject with mild changes in MT, nailfold capillary diameter, and skin pulse wave alteration. **G**-**L** The lower panels show an NTG patient with excessive changes in these parameters. **A**, **C**, **E**, **G**, **I**, and **K** show representative baseline images; other images are after CPT. For better visualization of the representative images, we increased brightness by 9% and contrast by 50% for **C**, **D**, **I**, and **J** using PowerPoint (Ver. 2304, Microsoft Corporation, Redmond, WA, USA)

Representative LSFG images, nailfold capillary diameter images, and graphs depicting skin pulse wave alterations during the protocol are presented in Fig. 4. The upper panels display a healthy subject with mild changes in MT, nailfold capillary diameter, and skin pulse wave alterations, while the lower panels exhibit an NTG patient with pronounced changes in these parameters.

## Discussion

In this study, we attempted to determine whether NTG patients had impaired vasoreactivity in response to cold stimulus, both systemically and locally in the eye. To do so, we established a CPT protocol combined with multimodal imaging that included LSFG, nailfold capillaroscopy, and skin blood flowmetry. We found different vasoreactive responses to cold provocation: there was a greater decrease in ONH-tissue BF and nailfold capillary diameter in NTG patients 4 min after the cold provocation and there was a greater increase in facial skin BF in NTG patients both 4 min and 6 min after the provocation. Additionally, we investigated the relationship between the percentage change in MT and the percentage changes in PA and nailfold capillary diameter. We found that the percentage change in MT was positively correlated with the percentage change in nailfold capillary diameter, but negatively correlated with the percentage change in PA. These results suggest that NTG patients might have abnormal vasoreactivity (i.e., they might have excessive vasoconstriction in the eye or nailfold and excessive vasodilation in the facial skin) in response to cold stimulus (i.e., sympathetic activation might occur).

We reconfirmed that there were nailfold capillary abnormalities in NTG patients at baseline. It has long been suggested that systemic vascular dysregulation may be involved in the pathophysiology of NTG [4, 35]. The nailfold capillaries have been the subject of research interest since the 1970s, possibly because of their easy accessibility and involvement in Raynaud’s phenomenon [36]. Although our study used different instruments and measured different parameters that were used in previous research, our finding that the nailfold capillaries were thinner and shorter in NTG patients is consistent with previous reports that nailfold capillary abnormalities were present in glaucoma patients [9, 37–39], indicating that we recruited a population of NTG patients with systemic vascular dysregulation.

During our CPT protocol, MOPP did not change in either the control or NTG groups, though IOP increased significantly in the control group. Considering that cold stress is considered to be a test of sympathetic nerve stimulation [22, 40, 41], which is thought to increase IOP [42], this result may be comprehensible. We also observed stable MOPP in the control subjects during CPT, despite incremental increases in IOP, which might have been due to sympathetic nerve activation and the tendency of BP to increase, though this effect did not reach statistical significance. The finding that IOP did not increase in the NTG group may be due to the possible effects of anti-glaucoma eye drops affecting the sympathetic pathway. Further investigation is needed to clarify this point. There is no consensus on how BP or IOP change during CPT, probably because past studies have used different CPT protocols, because of variations in BP response among individuals [43], and also because these measurements depend on the timing of the BP measurement. Kashima et al. reported that BP rose after 2 min, but then returned to baseline levels within 1 min [22]. In our protocol, the hemodynamics changes we observed in the ONH, nailfold, and facial skin were unlikely to have been due to changes in vital signs during and after the provocation, as there were no statistically significant changes in BP or MOPP.

In this study, the vascular responses of the ONH tissue and nailfold resembled each other, i.e., the capillaries in both these areas excessively contracted in NTG patients in response to CPT when compared with the control subjects. Previously, it was reported that CPT and nailfold capillaroscopy can be used to detect Raynaud’s phenomenon, which might be associated with NTG [12, 13, 36]. Our results support the idea that abnormal vasoreactivity in different body regions could be involved in the pathogenesis of NTG. Several reports have failed to show excessive contraction of the retinal or ophthalmic arteries [15, 17], while on the other hand, Gherghel et al. found that glaucoma patients showed a vasospastic response during CPT in the temporal ONH rim area [16]. Taken together with our results, the explanation for this discrepancy might be that capillaries originating from the short posterior ciliary artery can be affected by sympathetic excitation, because these arteries are innervated by autonomic nerves, while such innervation is absent or lacking in the retinal artery. Further investigation is needed to clarify this point in the future.

It is interesting to note that while the capillary hemodynamics of the ONH and nailfold were similar, facial skin BF showed opposite hemodynamics. In patients with Horner’s syndrome, the cheek on the side with sympathetic denervation shows diminished sweating and flushing [44]. Kashima et al. showed that facial skin BF increased in healthy subjects during CPT [22]. This suggests that the vasculature under the facial skin is innervated by the autonomic nervous system and dilates with sympathetic stimulation. The absence of such a response in the control subjects in this study may have been due to a shorter cold stimulus than that used by Kashima et al. (1 min vs. 2 min). Nevertheless, in the same condition, we found a stronger vasodilatory response in the NTG group. Regardless of whether sympathetic stimulation induces vasoconstriction or vasodilation in different body regions, we found excessive vasoreactivity in all of three regions in the NTG patients in this study. Furthermore, although ONH and nailfold observations are relatively easy to make, facial skin BF observations are even easier, in our experience. Thus, in the future, it might be possible to detect subjects with dysfunctional vasoregulation, and even glaucoma patients, simply by examining the facial skin BF reaction to CPT.

There are several limitations to this study. The most significant limitation is the small sample size, which was necessary for ethical reasons due to the burdensome nature of provocation testing. We chose the number of subjects by referring to previous studies that have used provocation testing; these studies generally included approximately 15 subjects per group [45–47]. The results in this study should be interpreted with great caution until we can verify them in a study with a larger sample size in the future. A second limitation is the potential bias arising from our study population. The subjects recruited in this study were not selected based on the presence of vasospastic abnormalities (e.g., having a history of cold hands), but the majority of open-angle glaucoma patients in Japan have normal-range IOP [48], meaning that they are more likely to have vascular abnormalities than the general population or high-tension glaucoma patients. In addition, it should be noted that we recruited only NTG patients with relatively mild visual field defects. This decision was made because if the patients had more severe glaucoma, the vessels might have been more atherosclerotic or even subject to drop-out [49–51], and we could not expect to clearly observe hyper-vasoreactivity. For this reason, we do not think that our results are generalizable to the overall population of patients with glaucoma. Third, we could not ask the NTG patients to discontinue the use of anti-glaucoma eye drops, again due to ethical considerations, so the differing vasoreactivity between the NTG and control groups might have been due to the effect of the eye drops. Indeed, only the glaucoma group received anti-glaucoma eye drops, so the difference in vasoreactivity might have been due to the presence or absence of eye drops. However, even when we divided the glaucoma group based on the use (*n* = 7) or non-use of β-blockers (*n* = 7), we did not observe a difference in percentage change in MT, nailfold capillary diameter, or PA (*P* = 0.443, *P* = 0.609, *P* > 0.999, respectively; Wilcoxon’s test). Thus, we still believe that the abnormality in vasoreactivity observed in the glaucoma patients was due to the presence or absence of glaucoma. In addition, since hyper-vasoreactivity was not only observed in the ONH but also in the nailfold and facial skin, we do not consider that the use of eye drops was a main cause. Furthermore, it is important to consider the potential influence of systemic vascular complications, such as diabetes mellitus. Thus, we added a history of diabetes mellitus (yes: 1; no: 0) and β-blocker use (yes: 1; no: 0) as explanatory variables in the linear mixed-effects models shown in Fig. 2 and confirmed that the results did not change. The results were as follows: the interaction term between the NTG group (reference, control group) and the 4-min protocol step (reference, baseline) significantly affected the changes in MT, nailfold capillary diameter, and PA (β = -9.51%, *P* = 0.017; β = -20.32%, *P* = 0.002; β =  + 18.08%, *P* = 0.017, respectively). Taken together, we believe that the difference in vasoreactivity in different body regions is mainly associated with the presence of glaucoma. Fourth, it is unclear at what depth the blood flowmetry device measures BF in each area. This uncertainty might affect our conclusions, because autonomic nerve innervation might differ at different depths of the tissue. Further investigation is needed to clarify these points in the future with multi-center studies with large sample sizes. The fifth limitation is that we analyzed the correlation of vasoreactivity in different body regions in data from a combined group of controls and glaucoma patients. In this study, we observed abnormal vasoreactivity in glaucoma patients that was not present in controls. In this case, if we separated the two groups for analysis, the values for vasoreactivity might cluster into normal and abnormal categories. Particularly with a small sample size, this could artificially result in a conclusion that there was no correlation in regional vascular reactivity. We believe that the crucial point is the reversal phenomenon, whereby despite the influence of the presence or absence of glaucoma, vasoreactivity showed a positive correlation in some regions (e.g., the ONH vs. the nailfold capillaries) and negative in others (e.g., the ONH vs. the facial skin). Therefore, we consider it worthwhile to present the analysis of the overall data. Nevertheless, we also consider that we might find weak correlations within each group if we had a larger sample size; we are eager to perform such an investigation in the future.

In conclusion, we found that a subgroup of NTG patients showed excessive vasoconstriction in the ONH tissue and nailfold capillaries and excessive vasodilation under the facial skin in response to cold-water provocation; this dysfunctional vasoregulation was revealed by a combination of CPT and simultaneous multimodal imaging analysis of the optic nerve head, nailfold, and facial skin BF.

## Supplementary Information

**Additional file 1.**

## Data Availability

The datasets used and/or analyzed during the current study are available from the corresponding author on reasonable request.

## Abbreviations

AL: Axial length
BCVA: Best-corrected visual acuity
BF: Blood flow
BP: Blood pressure
cpRNFLT: Circumpapillary retinal nerve fiber layer thickness
CPT: Cold provocation testing
CV: Coefficient of variation
DBP: Diastolic blood pressure
IOP: Intraocular pressure
logMAR: Logarithmic minimum angle of resolution
LSFG: Laser speckle flowgraphy
MBP: Mean blood pressure
MBR: Mean blur rate
MD: Mean deviation
MOPP: Mean ocular perfusion pressure
MT: Tissue-area mean blur rate
NTG: Normal-tension glaucoma
OCT: Optical coherence tomography
ONH: Optic nerve head
PA: Pulse wave amplitude
PR: Pulse rate
SBP: Systolic blood pressure
VF: Visual field
