# Supplementary figures and images for: Vasoreactivity of the optic nerve head, nailfold, and facial skin in response to cold provocation in normal-tension glaucoma patients
Source: BMC Ophthalmol. 2023 Jul 12;23:316. doi: 10.1186/s12886-023-03059-0 (PMC10339558; doi:10.1186/s12886-023-03059-0)

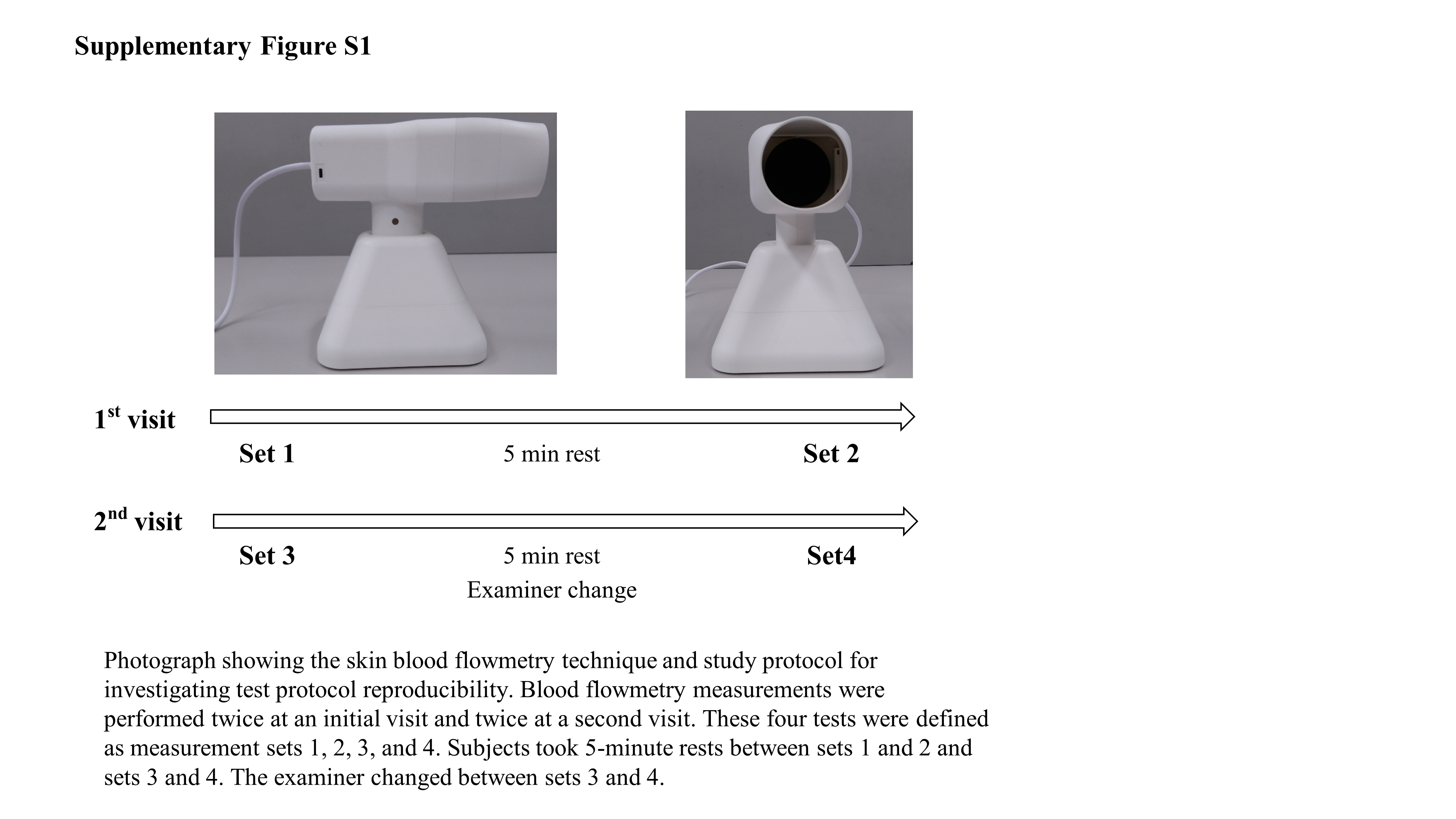

Supplement: Supplementary file 1 — Additional file 1. [file 12886_2023_3059_MOESM1_ESM.tif]
